# A Pelvic Mass in a Young Patient With Crohn's Disease

**DOI:** 10.14309/crj.0000000000001627

**Published:** 2025-02-26

**Authors:** Charles D. Evers, Aishwarya Ravindran, Frida Rosenblum, Frederick Weber

**Affiliations:** 1 Department of Medicine, Division of Internal Medicine, Heersink School of Medicine, University of Alabama at Birmingham, Birmingham, AL; 2 Department of Pathology, Heersink School of Medicine, University of Alabama at Birmingham, Birmingham, AL; 3 Department of Medicine, Division of Gastroenterology and Hepatology, Heersink School of Medicine, University of Alabama at Birmingham, Birmingham, AL

**Keywords:** Crohn's disease, Castleman disease, inflammatory bowel disease, lymphoproliferative

## Abstract

Castleman disease (CD) is a rare group of lymphoproliferative disorders subdivided based on clinical features. Although not fully understood, the pathogenesis of both CD and Crohn's disease involves a combination of immune dysregulation and infectious and environmental factors. Interleukin-6, a proinflammatory cytokine, is associated with both diseases and can serve as a common therapeutic target in CD. We report a rare case of coexisting unicentric Castleman disease in a young patient with Crohn's disease.

## INTRODUCTION

Castleman disease (CD) is a group of lymphoproliferative disorders that share common morphological features on lymph node biopsy and is further classified based on clinical features into unicentric Castleman disease (UCD) or multicentric Castleman disease (MCD).^1^ UCD is the most common variant and typically presents as a solitary mass without systemic symptoms. Surgical excision of the affected lymph node region is diagnostic and therapeutic with a low recurrence rate. MCD affects multiple lymph nodes and tissues and typically presents with constitutional symptoms (fever, night sweats, anasarca, and weight loss) and generalized lymphadenopathy with splenomegaly. MCD is further subdivided into idiopathic MCD (iMCD) and Kaposi sarcoma herpesvirus-associated MCD. Although the pathogenesis of CD is poorly understood, data suggest that UCD is a clonal neoplastic process arising from stromal cells, and several associations exist with other autoimmune diseases (Table 1).^2,8^ Studies show interleukin-6 (IL-6) plays a role in subsets of CD (particularly iMCD), and anti-IL-6 monoclonal antibodies (mAb) are the first-line therapy for symptomatic patients; however, about half of patients do not respond to IL-6 inhibition, suggesting alternative cytokines are involved.^10^ Interestingly, IL-6 is also implicated in the pathogenesis of Crohn's disease.^4,5^ However, the infrequency of reported cases of patients with both conditions suggests other factors may be involved. This case provides the third description of Crohn's disease coexisting with CD (Table 2).^6,7^

**Table 1.** Autoimmune associations with CD

Autoimmune condition	Association with CD
Systemic lupus erythematosus	Both commonly present with localized or generalized lymphadenopathy, systemic symptoms^2,3^
Rheumatoid arthritis	Lymphadenopathy, pathogenesis involves dysregulation of IL-6^2,4^
Crohn's disease	Pathogenesis involves dysregulation of IL-6^1,4–7^
Sjogren syndrome	Both associated with anti-Sjogren-syndrome-related antigen A antibodies^2^
Adult-onset still's disease	Similar histopathological findings^2^
Paraneoplastic pemphigus	Reported in patients with CD; UCD > iMCD^2,8^
Psoriasis	Reported in patients with iMCD^8^
Myasthenia gravis	Reported in patients with iMCD^8^
Autoimmune hemolytic anemia	Diagnosed in 30%–40% of patients with iMCD at initial presentation^8^
Autoimmune thrombocytopenia	Diagnosed in 15%–20% of patients with iMCD at initial presentation^8^
Evan syndrome	Reported in patients with iMCD^8,9^

Table 1 includes autoimmune conditions associated with CD. While these associations exist, the exact relationship between CD and autoimmune disorders is not fully understood. This list is not exhaustive.

CD, Castleman disease; IL-6, interleukin-6; iMCD, idiopathic multicentric Castleman disease; UCD, unicentric Castleman disease.

**Table 2.** Case reports with CD and Crohn's disease

Case report	Age (y)	Sex	Location of CD	Diagnosis
Gupta A, et al^6^	51	Female	Retroperitoneal Mass (5.7 × 3.5 × 2.0 cm)	Unicentric CD, hyaline vascular variant
Gupta R, et al^7^	25	Male	Mesenteric lymph nodes	Multicentric CD, hyaline vascular variant
Evers, et al	32	Female	Retroperitoneal Mass (4.3 × 5.7 × 5.1 cm)	Unicentric CD, hyaline vascular variant

CD, Castleman disease.

## CASE REPORT

A 32-year-old woman presented with left lower quadrant abdominal pain for 3 weeks. She had a medical history of Crohn's disease, endometriosis, disseminated histoplasmosis, and nephrolithiasis. The pain was intermittent, dull, and radiated to the left flank. Her Crohn's disease had been clinically quiescent for several years, managed at another institution on mesalamine, and she denied recent fevers, night sweats, weight loss, diarrhea, or rectal bleeding. The pain was not associated with oral intake, and her stools were well formed. Further review of systems was entirely negative.

The patient was diagnosed with Crohn's disease 13 years before presentation (2010). At that time, 75 cm of the jejunum and 10 cm of the terminal ileum, including the ileocecal valve, were surgically resected at an outside facility. She was discharged on mesalamine and sporadically adhered to this regimen; 3 years later (2013), she developed a small bowel obstruction due to a duodenal stricture that required a lengthy hospitalization included jejunal tube placement for nutrition, initiation of adalimumab and methotrexate, and multiple balloon dilations of the duodenal stricture. She remained on methotrexate and adalimumab with excellent disease control for 4 years, but in 2017, she was hospitalized with disseminated histoplasmosis. Methotrexate and adalimumab were transitioned to mesalamine during and after histoplasmosis treatment. Since 2017, her Crohn's disease remained well controlled without other therapy.

**Figure 1.**
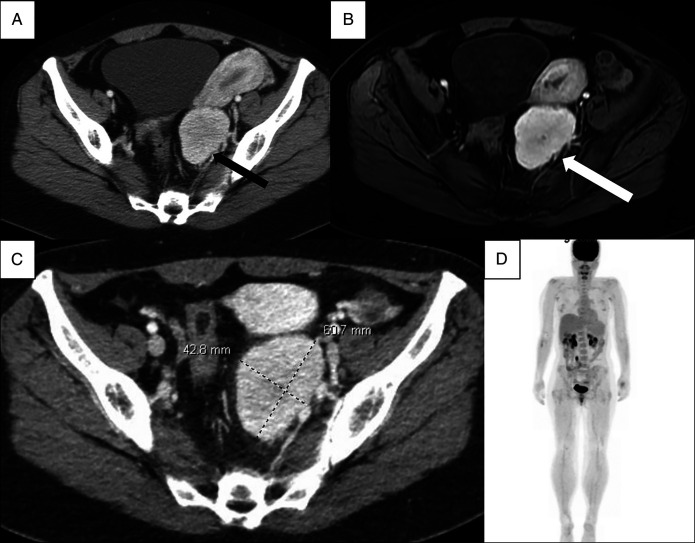
Radiographic imaging. Radiologically, the differential diagnosis included indolent lymphoma, paraganglioma, nerve sheath tumor, desmoid, or histoplasmosis recurrence. (A) Contrast-enhanced computed tomography, September 2019, (B) magnetic resonance of the abdomen—T1-weighted postcontrast, December 2022, (C) contrast-enhanced computed tomography, December 2023, (D) positron emission tomography scan, February 2024.

In 2019, she first experienced left lower quadrant pain, and computed tomography (CT) imaging revealed a 5.3 cm × 3.8 cm heterogeneous solid mass (Figure 1). Diagnostic laparoscopy soon after showed grade I endometriosis and a retroperitoneal mass. Further evaluation was planned but was disrupted due to the pandemic.

Physical examination was notable only for mild suprapubic and left lower quadrant tenderness without palpable lymphadenopathy or masses. Notable initial laboratory studies are included in Table 3.

**Table 3.** Notable laboratory values at initial presentation

Test	Result	Reference range
Hemoglobin (g/dL)	12.7	11.3–15.2
White blood cell count (×10^9^/L)	5.0	4.0–11.0
Platelet count (×10^9^/L)	305	150–400
Neutrophils (%)	85	35–73
Lymphocytes (%)	8	15–52
C-reactive protein (mg/L)	3.23	0–10.9

**Figure 2.**
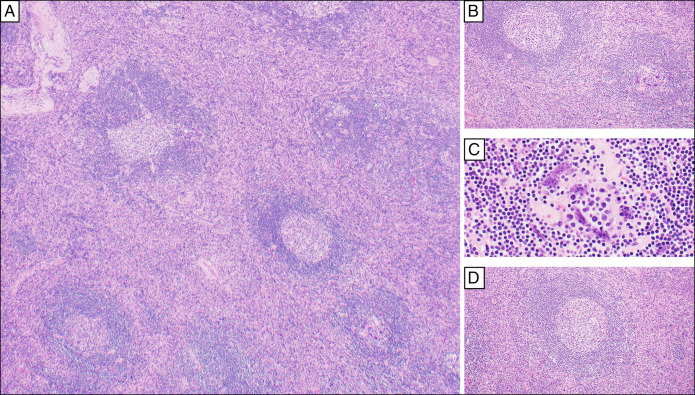
Histology of the pelvic soft tissue mass. Microscopic evaluation of the specimen on hematoxylin and eosin-stained sections shows portions of lymphoid tissue with somewhat distorted nodal architecture. The lymphoid parenchyma (A, 4×) shows follicles of varying sizes with a somewhat expanded interfollicular area (B, 10×), demonstrating the proliferation of small, hyalinized vessels in a heterogeneous background of small mature lymphocytes, histiocytes, occasional immunoblasts, and a few plasma cells. Dysplastic follicular dendritic cells characterized by hyperchromatic nuclei with multinucleation are present (C, 40×). Some of the follicles demonstrate twinning, regressive changes, lymphocyte depletion, hyaline deposits, and radially penetrating capillaries, surrounded by concentric layers of mantle zone lymphocytes (A 4×; D 20×).

**Figure 3.**
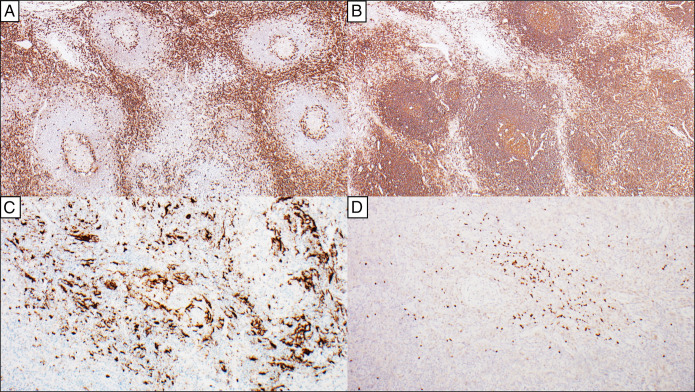
Immunophenotypic characterization of the pelvic soft tissue mass. The interfollicular areas are composed of CD3-positive T cells (A, 4×) that are cytologically unremarkable. The lymphoid follicles are composed of CD20-positive B cells (B, 4×). CXCL13 (C, 10×) highlights the FDC meshworks, including the dysplastic FDCs, and these demonstrated coexpression of other FDC markers (CD21 and clusterin (not shown)). Patchy foci in the interfollicular areas are composed of indolent TdT-positive and CD3-positive T lymphoblasts (D, 10×), which are known to be associated with Castleman disease. FDC, follicular dendritic cell.

Magnetic resonance enterography revealed a well-defined, lobulated, avidly enhancing left pelvic sidewall lesion measuring 4.3 cm × 5.7 cm × 5.1 cm felt radiographically consistent with a paraganglioma, peripheral nerve sheath tumor, or desmoid tumor (Figure 1), and subsequent CT revealed similar findings (Figure 1). No other lesions were noted by radiologic examination (Figure 1). Flexible sigmoidoscopy with endoscopic ultrasound-guided biopsy noted a polymorphous population of lymphocytes, but no definitive diagnosis was ascertained. Due to ongoing discomfort, she elected to undergo surgical resection of the mass. She tolerated the procedure well with no complications; histology of the mass is provided in Figures 2 and 3. The overall histopathologic features and radiologic findings were diagnostic of unicentric Castleman disease, the hyaline vascular variant. A comprehensive next-generation sequencing analysis was negative for pathogenic mutations and fusions.

## DISCUSSION

UCD usually presents as an enlarging mass, which may be discovered by visualization, palpation, or incidentally found on radiographical imaging. Although some cases are asymptomatic, the most common presenting symptom is pain secondary to the mass effect, which disrupts local structures and compresses nerves. Diagnosis requires surgical excision and histological examination of the entire lymph node. As demonstrated in this case, core needle biopsy/fine-needle aspirate is usually insufficient to confirm the diagnosis of CD because macroscopic architectural features of the lymph node are usually needed for diagnosis. Including a whole-body CT or fluorodeoxyglucose-positron emission tomography may be necessary to evaluate for other lymphoproliferative disorders. Complete surgical excision is curative in a majority of cases of UCD, and overall survival at 5 years exceeds 90%.^1^

This is the third case report describing concomitant CD and Crohn's disease. As previously mentioned, IL-6 is implicated in the pathogenesis of each, although increased levels are more closely associated with iMCD than UCD. Siltuximab, an anti-IL-6 mAb, is the first-line treatment and only US Food and Drug Administration-approved therapy for iMCD.^11^ IL-6 mAbs (tocilizumab, PF-04236921) have been studied for use in Crohn's disease and demonstrate efficacy; however, an association with increased risk of gastrointestinal abscess/perforation and profound immunosuppression has limited their development.^12–14^ Olamkicept, a fusion protein that inhibits IL-6 trans-signaling, selectively targets the IL-6 pathway responsible for chronic inflammation while sparing the classic IL-6 signaling pathway responsible for intestinal barrier integrity, immune homeostasis, and mucosal regeneration.^5^ According to early studies, olamkicept effectively reduces inflammation and provides clinical remission of inflammatory bowel disease for some patients without an increased risk of perforation.^15,16^ The IL-6 pathway represents a promising therapeutic target for emerging therapies in inflammatory bowel disease.

Although the patient's IL-6 levels are normal at present, her level at the time of active Crohn's disease or prior disseminated histoplasmosis is unknown as is whether either played a role in the development of CD. She was not treated with any anti-IL-6 therapies because she lacked systemic symptoms and radiographic evidence to suggest iMCD. Whether chronic immune stimulation in Crohn's disease is the cause of the increased risk of lymphoma and possibly CD in Crohn's disease is unclear.^17^

## DISCLOSURES

Author contributions: C.D.E.: Primary composer, article guarantor; reviewed case, performed literature reviews, composed first draft, composed revisional draft, corresponding author. A.R., F.R.: Worked together to prepare the pathological report, make the diagnosis, photographed the gross pathology, composed Figures 2 and 3, and provided the legend text for Figures 2 and 3. Both authors reviewed the first draft and revisional draft. F.W.: primary editor, senior author; provided clinical care for the patient. Reviewed the first draft, provided recommendations for revisions, reviewed revisional draft, performed role of senior author.

Financial disclosure: None to report.

Informed consent was obtained for this case report.
